# Causality of anti-*Helicobacter pylori* IgG levels on myocardial infarction and potential pathogenesis: a Mendelian randomization study

**DOI:** 10.3389/fmicb.2023.1259579

**Published:** 2023-09-14

**Authors:** Qiubo Wang, Yingbo Liu, Zhenxing Xu, Zhimiao Wang, Mei Xue, Xinran Li, Ye Wang

**Affiliations:** ^1^Department of Cardiology, The First Affiliated Hospital of Shandong First Medical University & Shandong Provincial Qianfoshan Hospital, Shandong Medicine and Health Key Laboratory of Cardiac Electrophysiology and Arrhythmia, Jinan, China; ^2^Shandong First Medical University & Shandong Academy of Medical Sciences, Jinan, China; ^3^Center for Reproductive Medicine, Shandong University, Jinan, China; ^4^Key Laboratory of Reproductive Endocrinology of Ministry of Education, Shandong University, Jinan, China

**Keywords:** anti-*Helicobacter pylori* IgG levels, myocardial infarction, HDL cholesterol, causal association, Mendelian randomization

## Abstract

**Background:**

Previous observational studies have shown that a potential relationship between anti-*Helicobacter pylori* (*H. pylori*) IgG levels and Myocardial Infarction (MI). Nevertheless, the evidence for the causal inferences remains disputable. To further clarify the relationship between anti-*H. pylori* IgG levels and MI and explore its pathogenesis, we conducted a Mendelian randomization (MR) analysis.

**Methods:**

In this study, we used two-sample Mendelian Randomization (MR) to assess the causality of anti-*H. pylori* IgG levels on MI and potential pathogenesis, 12 single nucleotide polymorphisms (SNPs) related to anti-*H. pylori* IgG levels were obtained from the European Bioinformatics Institute (EBI). Summary data from a large-scale GWAS meta-analysis of MI was utilized as the outcome dataset. Summary data of mediators was obtained from the FinnGen database, the UK Biobank, the EBI database, MRC-IEU database, the International Consortium of Blood Pressure, the Consortium of Within family GWAS. Inverse variance weighted (IVW) analysis under the fixed effect model was identified as our main method. To ensure the reliability of the findings, many sensitivity analyses were performed.

**Results:**

Our study revealed that increases of anti-*H. pylori* IgG levels were significantly related to an increased risk of MI (OR, 1.104; 95% CI,1.042–1.169; *p* = 7.084 × 10^−4^) and decreases in HDL cholesterol levels (*β*, −0.016; 95% CI, −0.026 to −0.006; *p* = 2.02 × 10^−3^). In addition, there was no heterogeneity or pleiotropy in our findings.

**Conclusion:**

This two-sample MR analysis revealed the causality of anti-*H. pylori* IgG levels on MI, which might be explained by lower HDL cholesterol levels. Further research is needed to clarify the results.

## Introduction

Cardiovascular diseases have become one of the leading causes of death in adults in recent decades due to an increase in their incidence and prevalence (Vaughan et al., 2015; Widmer et al., 2015). Myocardial infarction (MI) is an acute and severe cardiovascular disease, that is brought on by ischemia of the heart muscle and blockage of the coronary arteries, poses a significant threat to patients’ lives, and has become a serious public health problem (Lu et al., 2015). MI is caused by a variety of factors, including lifestyle, diet, genetics, and environmental factors (Anand et al., 2008; Dahabreh and Paulus, 2011; Finegold et al., 2013). Reduced modifiable risk factors can improve MI prevention and control (Teo et al., 2009; Colombo et al., 2014; Smyth et al., 2016), which has important public health implications. There is evidence to suggest that inflammation is a key factor in the development and progression of atherosclerosis (Ballantyne and Nambi, 2005). Chronic infection with various pathogens may trigger inflammatory responses in blood vessel walls, which may be crucial in the development of atherosclerosis and the progression of coronary heart disease (CHD) (Epstein, 2002; Zhu and Liu, 2020; Jung and Lee, 2022).

*Helicobacter pylori* (*H. pylori*) is a spiral, gram-negative microaerobic bacteria that has infected about half of the world’s population (Kotilea et al., 2019; Choi et al., 2020). There are a variety of symptoms resulting from *H. pylori* colonization, including gastritis, peptic ulcers, and neoplastic disease (Peek and Blaser, 2002; Buti et al., 2020; Holleczek et al., 2020). In recent years, a large number of observational studies have indicated that chronic *H. pylori* infection is concerned with cardiovascular and cerebrovascular diseases, such as cardiovascular disease, thrombotic cerebrovascular disease, and peripheral vascular disease (de Luis et al., 1998; Lee et al., 2018; Wan et al., 2018). The earliest evidence between *H. pylori* infection with CHD was proposed by Mendall et al. (1994). During the past decade, researchers have examined the association between *H. pylori* seropositivity and CHD using epidemiological methods. Several studies have shown a potential relationship between *H. pylori* seropositivity and CHD (Park et al., 2011; Shmuely et al., 2014). However, numerous studies showed contradictory results on the role of *H. pylori* seropositivity in CHD (Al-Nozha et al., 2003; Rothenbacher et al., 2003). Of course, there may be many unknown confounding factors affecting the robustness of the results. Thus, whether *H. pylori* plays a causal role in the MI remains undiscerned. It is urgent to further clarify the relationship between *H. pylori* infection and MI and explore its pathogenesis. The underlying mechanisms between *H. pylori* infection and MI remain unclear. A substantial quantity of epidemiologic and clinical evidence addressing connections between *H. pylori* infection and risk factors for MI has been revealed during recent decades. Firstly, a study conducted by Gunji et al. showed that *H. pylori* seropositivity was significantly associated with higher systolic blood pressure, lower HDL cholesterol levels, and higher LDL cholesterol levels (Gunji et al., 2008). Secondly, Chen TP et al. revealed that *H. pylori*-infected individuals had significantly higher body mass index and fasting glucose in cross-sectional research including 3,578 subjects (Chen et al., 2015). Then, a study concluded that there was a remarkable relationship between chronic *H. pylori* infection and high levels of HbA1c and decreased insulin secretion (Hsieh et al., 2013). Furthermore, *H. pylori* infection has been revealed to be associated with vitamin deficiency (Franceschi et al., 2014). Vitamin levels play an important role in CHD. Finally, inflammation reactions can be triggered by *H. pylori*. High levels of the inflammatory cytokines IL-6 and tumor necrosis factor have been related to *H. pylori* infection in individuals with CHD (Schumacher et al., 2002).

MR analysis is applied to investigate the relationship between exposure and outcome, which can provide robust causality by utilizing one or multiple genetic variants, such as single nucleotide polymorphisms (SNPs) (Smith and Ebrahim, 2004; Lawlor et al., 2008). The MR study was built on the Mendelian inheritance rule, which states that the parents’ genetic alleles are randomly dispersed to the descendants during the process of meiosis, which is supposed to be equivalent to RCT. Using MR, these biases that are common in observational studies can be avoided by utilizing genetic variables reflecting exposure to verify the causal association of the risk variables connected to the disease (Smith and Ebrahim, 2003, 2004). Serum immunoglobulin G antibody to *H. pylori* is most widely adopted in population-based research, and its accuracy has been shown in several studies across a wide variety of ethnic groups and nations (Feldman and Evans, 1995; Roberts et al., 2000). A two-sample MR analysis was performed in the present study, hoping to clarify their causal relationship, explore its pathogenesis, and provide useful advice for clinical practice.

## Methods

### Mendelian randomization design

**Figure 1:**
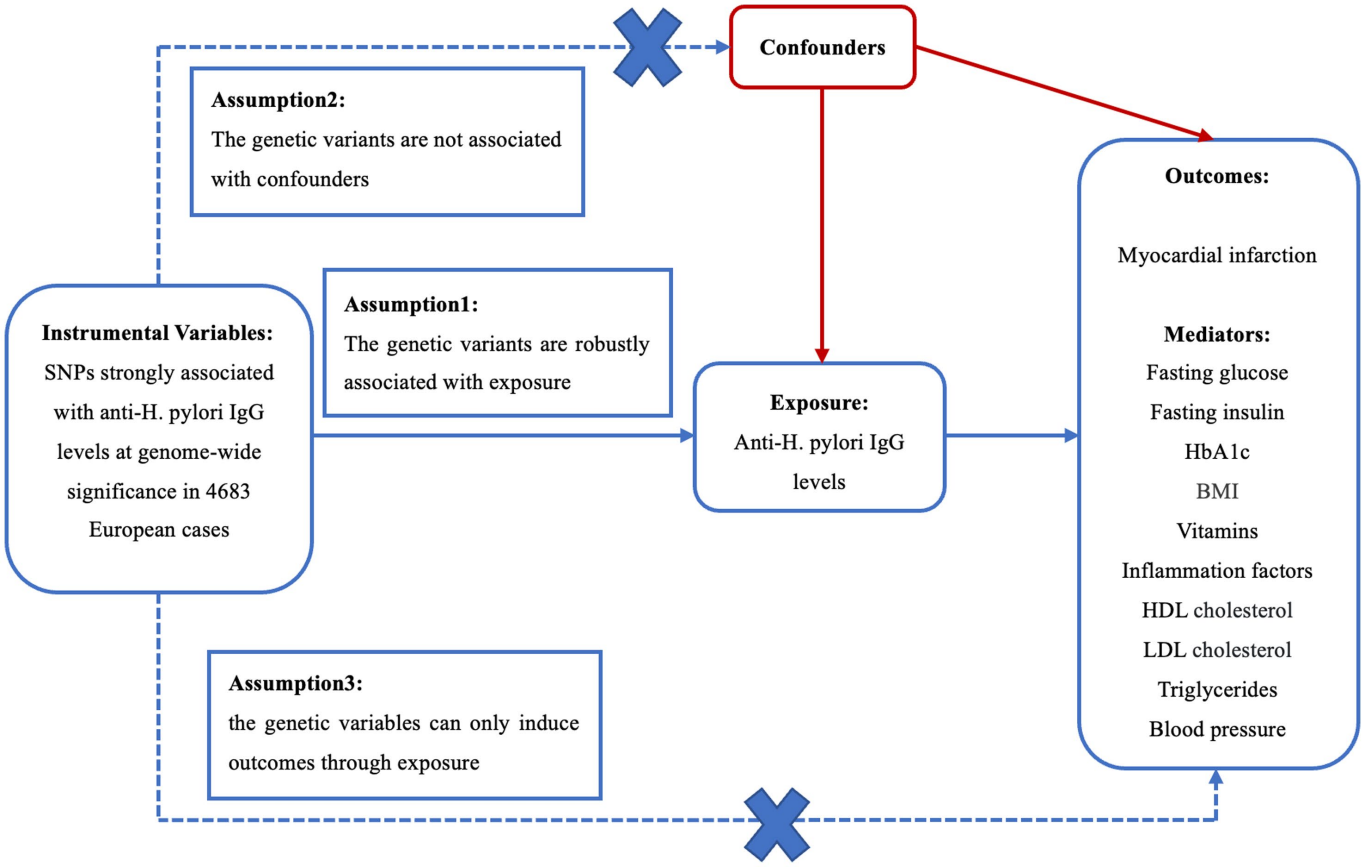
Three crucial hypotheses of the Mendelian randomization study. SNPs, single-nucleotide polymorphisms; *H. pylori*, *Helicobacter pylori*; LDL, low density lipoprotein; HDL, high density lipoprotein; BMI, body mass index.

A MR study was conducted to evaluate the causality of anti-*H. pylori* IgG levels on MI and mediators. There are three core assumptions for determining the genetic instrumental variables (IVs) that are at the centre of the MR analysis (Sekula et al., 2016). First, the genetic instruments should be strongly concerned with anti-*H. pylori* IgG levels. Second, there is no connection between the SNPs and potential confounders. Third, IVs can only induce outcomes through anti-*H. pylori* IgG levels (Figure 1).

### Data sources

From the publicly available data source maintained by the European Bioinformatics Institute (EBI), we acquired the GWAS summary statistics for Anti-*H. pylori* IgG levels at https://gwas.mrcieu.ac.uk/datasets/ieu-b-4905/, which included 4,683 European cases. The MI GWAS summary dataset was obtained from the GWAS conducted by Hartiala et al. (2021), which contained 395,795 participants from Europe, both male and female. Possible pathogenesis underlying the association between *H. pylori* and MI includes fasting glucose, HbA1c, fasting insulin, body mass index (BMI), lipid traits, inflammation factors, vitamins, and blood pressure. The GWAS summary statistics for fasting glucose and fasting insulin were obtained from the study conducted by Chen et al. (2021). HbA1c was obtained from the Consortium of Within family GWAS. The GWAS summary statistics for inflammation factors were collected from the EBI database. The GWAS summary statistics for BMI and vitamin were accessible in the MRC Integrative Epidemiology Unit (MRC-IEU) database. The GWAS summary statistics for lipid traits were available from the United Kingdom Biobank database, containing triglyceride, LDL cholesterol, and HDL cholesterol levels (Richardson et al., 2020). The GWAS summary statistics for blood pressure were accessible to the International Consortium of Blood Pressure, including diastolic blood pressure and systolic blood pressure. The details of the GWAS data included in this study are shown in Table 1.

**Table 1:** Details of the GWAS data in this study.

Phenotype	Data source	Ethnicity	Sample size	Web source
Anti-*H. pylori* IgG	Chong A et al.	European	4,683	https://gwas.mrcieu.ac.uk/datasets/ieu-b-4,905/
Myocardial infarction	Hartiala et al.	European	395,795	https://gwas.mrcieu.ac.uk/datasets/ebi-a-GCST011364/
Fasting glucose	Chen et al.	European	200.622	https://gwas.mrcieu.ac.uk/datasets/ebi-a-GCST90002232/
Fasting insulin	Chen et al.	European	151.013	https://gwas.mrcieu.ac.uk/datasets/ebi-a-GCST90002238/
HbA1c	Within family GWAS consortium	European	45,734	https://gwas.mrcieu.ac.uk/datasets/ieu-b-4842/
BMI	MRC-IEU	European	461,460	https://gwas.mrcieu.ac.uk/datasets/ukb-b-19953/
Vitamin C	MRC-IEU	European	460,351	https://gwas.mrcieu.ac.uk/datasets/ukb-b-15175/
Vitamin D	MRC-IEU	European	460,351	https://gwas.mrcieu.ac.uk/datasets/ukb-b-12648/
Vitamin B12	MRC-IEU	European	64,979	https://gwas.mrcieu.ac.uk/datasets/ukb-b-19524/
Interleukin-18	EBI database	European	21,758	https://gwas.mrcieu.ac.uk/datasets/ebi-a-GCST90012024/
Interleukin-6	EBI database	European	21,758	https://gwas.mrcieu.ac.uk/datasets/ebi-a-GCST90012005/
Interleukin-8	EBI database	European	21,758	https://gwas.mrcieu.ac.uk/datasets/ebi-a-GCST90011994/
Interleukin-4	EBI database	European	8,124	https://gwas.mrcieu.ac.uk/datasets/ebi-a-GCST004453/
Interleukin-10	EBI database	European	7,681	https://gwas.mrcieu.ac.uk/datasets/ebi-a-GCST004444/
TNF-α	EBI database	European	3,454	https://gwas.mrcieu.ac.uk/datasets/ebi-a-GCST004426/
HDL cholesterol	UK Biobank	European	403,943	https://gwas.mrcieu.ac.uk/datasets/ieu-b-109/
LDL cholesterol	UK Biobank	European	440,546	https://gwas.mrcieu.ac.uk/datasets/ieu-b-110/
Triglycerides	UK Biobank	European	441,016	https://gwas.mrcieu.ac.uk/datasets/ieu-b-111/
Diastolic blood pressure	International Consortium of Blood Pressure	European	757,601	https://gwas.mrcieu.ac.uk/datasets/ieu-b-39/
Systolic blood pressure	International Consortium of Blood Pressure	European	757,601	https://gwas.mrcieu.ac.uk/datasets/ieu-b-38/

*H. pylori*, *Helicobacter pylori*; LDL, low density lipoprotein; HDL, high density lipoprotein; BMI, body mass index; TNF-α, Tumor necrosis factor alpha.

### Selection and validation of SNPs

After we set the threshold of the *p* value as 5 × 10^−8^, we did not obtain any independent SNPs. In order to contain more SNPs that concerned with anti-*H. pylori* IgG levels, we used a more lenient criterion (*p* < 5 × 10^−6^) which had been applied to previous MR research (Ong and MacGregor, 2019). At the genome-wide significance level (*p* < 5 × 10^−6^), we discovered 12 single nucleotide polymorphisms (SNPs) related to anti-*H. pylori* IgG levels. Effective MR analyses require no linkage disequilibrium (*r*^2^ < 0.001) across specific SNPs (Abecasis et al., 2010). Consequently, 12 distinct SNPs connected to anti-*H. pylori* IgG levels were determined. In addition, when the F-statistic is greater than 10, the SNPs were regarded as adequate to moderate the effect of potential bias, using the following formula: *F* = *R*^2^ × (*N*−2) / (1−*R*^2^), *R*^2^ = 2 × (1−MAF) × (MAF) × *β*^2^ (Burgess and Thompson, 2011). The F-statistics varied from 26 to 33, greater than the traditional level of 10. Details of 12 SNPs can be found in the Supplementary material.

### Statistical analysis

First, the SNPs for exposure and outcome were harmonized to coordinate allelic directions and eliminate palindromic sequences. Then we deleted SNPs that were strongly related to the outcomes. In this study, no SNPS were eliminated in this step. Before we conducted MR analysis, we detected outliers using the MR-PRESSO method to enhance the robustness of the results. As a primary analysis, we used inverse variance weighted (IVW) analysis under the fixed effect model as our main method because no heterogeneity was found in our study. Additionally, to ensure the results are robust, multiple complementary analyses were conducted, like IVW under the random effects model, weighted median, and MR-egger. In order to verify the reliability of the primary results, sensitivity analysis has been crucial for identifying underlying pleiotropy and heterogeneity in MR estimates. Pleiotropic bias was assumed to exist if the MR-Egger intercept had the *p*-value of less than 0.05 (Bowden et al., 2015). Moreover, we used the MR-PRESSO method to detect outliers before MR estimates were proceeded. MR-PRESSO eliminated abnormal SNPs (outliers) to detect potential horizontal pleiotropic and test whether there is a difference between the results before and after correction (Verbanck et al., 2018). The leave-one-out method was applied to analyze the sensitivity of the results by sequentially removing one SNP at a time to examine whether a single SNP with a large horizontal pleiotropy effect might affect the MR estimates. In this research, Cochran’s Q was computed to examine the heterogeneity brought on by various SNPs. A total of MR analyses was performed using the R package “TwosampleMR.”

## Results

### Causal effects of anti-*H. pylori* IgG levels on MI

**Figure 2:**
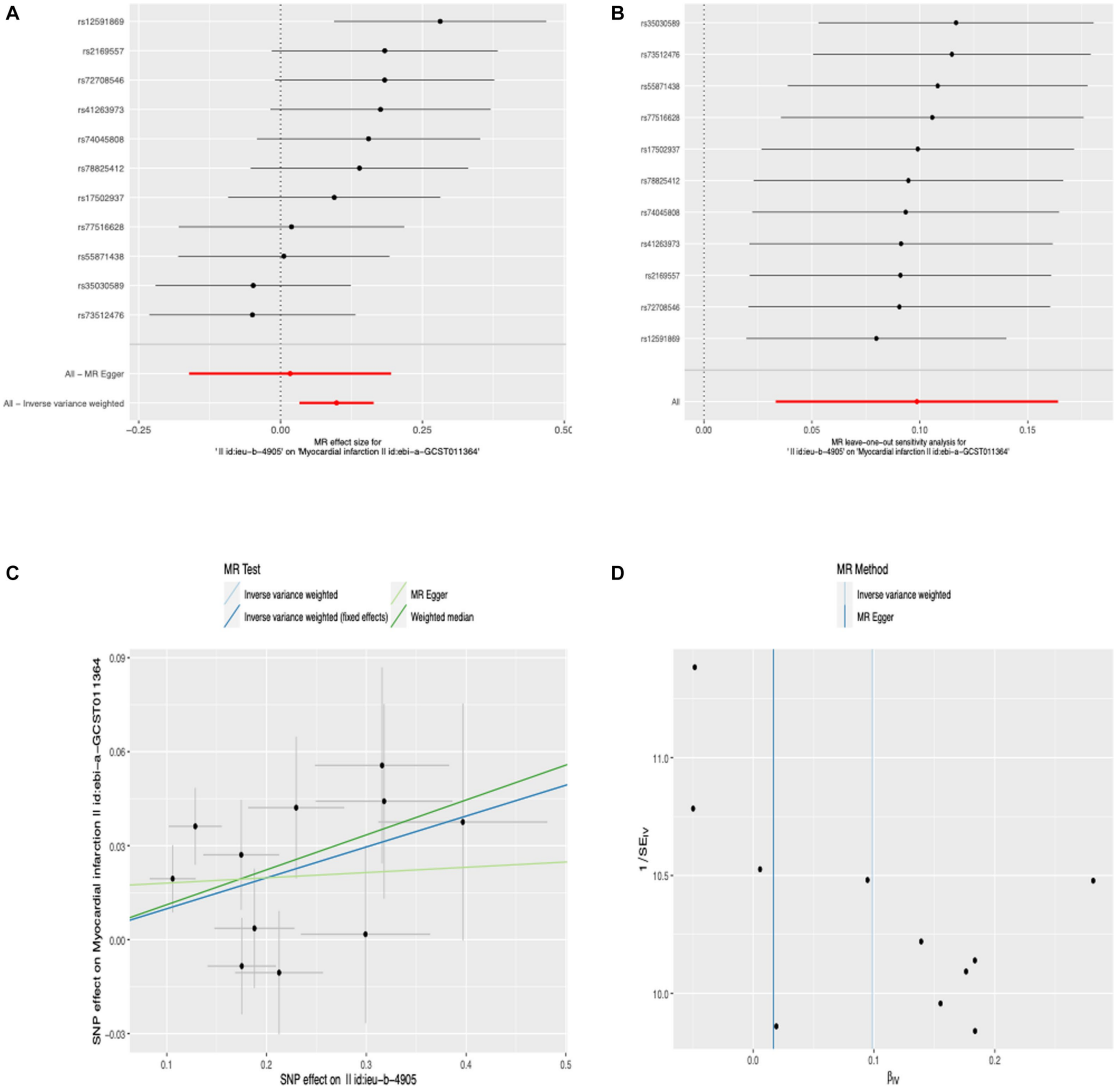
Forest plot **(A)**, sensitivity analysis **(B)**, scatter plot **(C)**, and funnel plot **(D)** of the effect of anti-*H. pylori* IgG levels on MI.

Before we conducted MR estimates, an outlier (rs117912702) with large pleiotropy was detected by MR-PRESSO analysis, so we got rid of it. We finally selected 11 SNPs for this study. According to the IVW analysis under fixed effect, there was a significant association between anti-*H. pylori* IgG levels and MI (OR, 1.104; 95% CI, 1.042–1.169; *p* = 7.084 × 10^−4^). Similar risk estimates were obtained using IVW analysis under the random effect approach (OR, 1.104; 95% CI, 1.034–1.178; *p* = 3.116 × 10^−3^) and the weighted median (OR, 1.178; 95% CI, 1.029–1.214; *p* = 8.143 × 10^−3^). The approaches of the MR-Egger regression could not obtain this result (Figure 2A). We then used sensitivity analyses to check the reliability of our results. After excluding the pleiotropic variant, no horizontal pleiotropy was found by the MR-PRESSO method. For all outcomes, according to the MR-Egger regression, there did not appear to be horizontal pleiotropy based on the intercept term (intercept = 0.016, SE = 0.017, *p* = 0.358) (Figure 2C). We used the leave-one-out study to test the robustness of the results. All error lines are to the left of 0, indicating that the results are reliable and demonstrating that there are no SNPs with a large horizontal pleiotropic effect (Figure 2B). Then, to test the heterogeneity of the study, the Cochran Q-test derived *p* value as 0.22 of MR-Egger and *p* value as 0.22 of IVW. In general, there is no heterogeneity in this study (Figure 2D). Details of the MR estimates and sensitivity analyses can be found in the Supplementary material.

### Causal effects of anti-*H. pylori* IgG levels on potential pathogenesis

**Figure 3:**
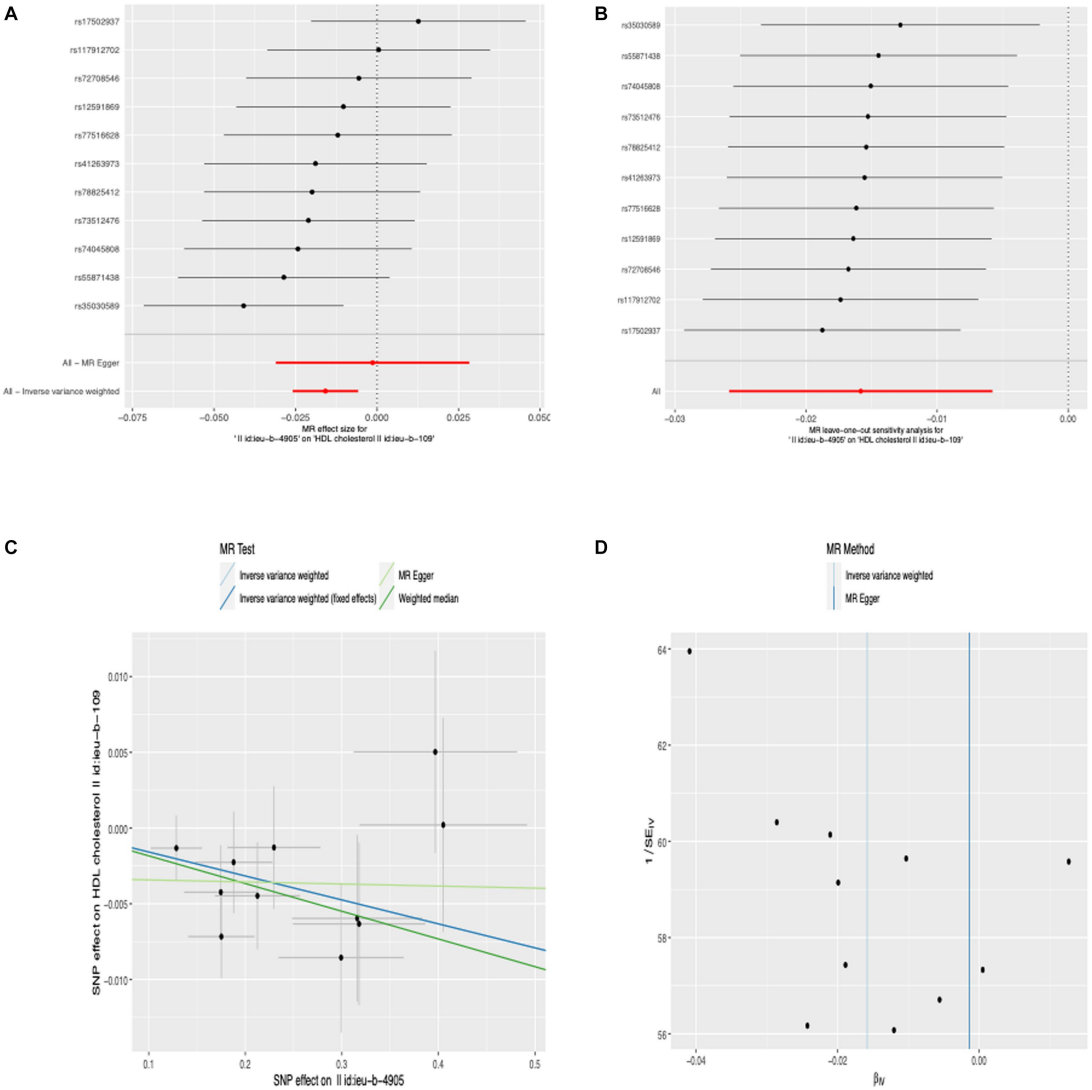
Forest plot **(A)**, sensitivity analysis **(B)**, scatter plot **(C)**, and funnel plot **(D)** of the effect of anti-*H. pylori* IgG levels on HDL cholesterol levels.

**Figure 4:**
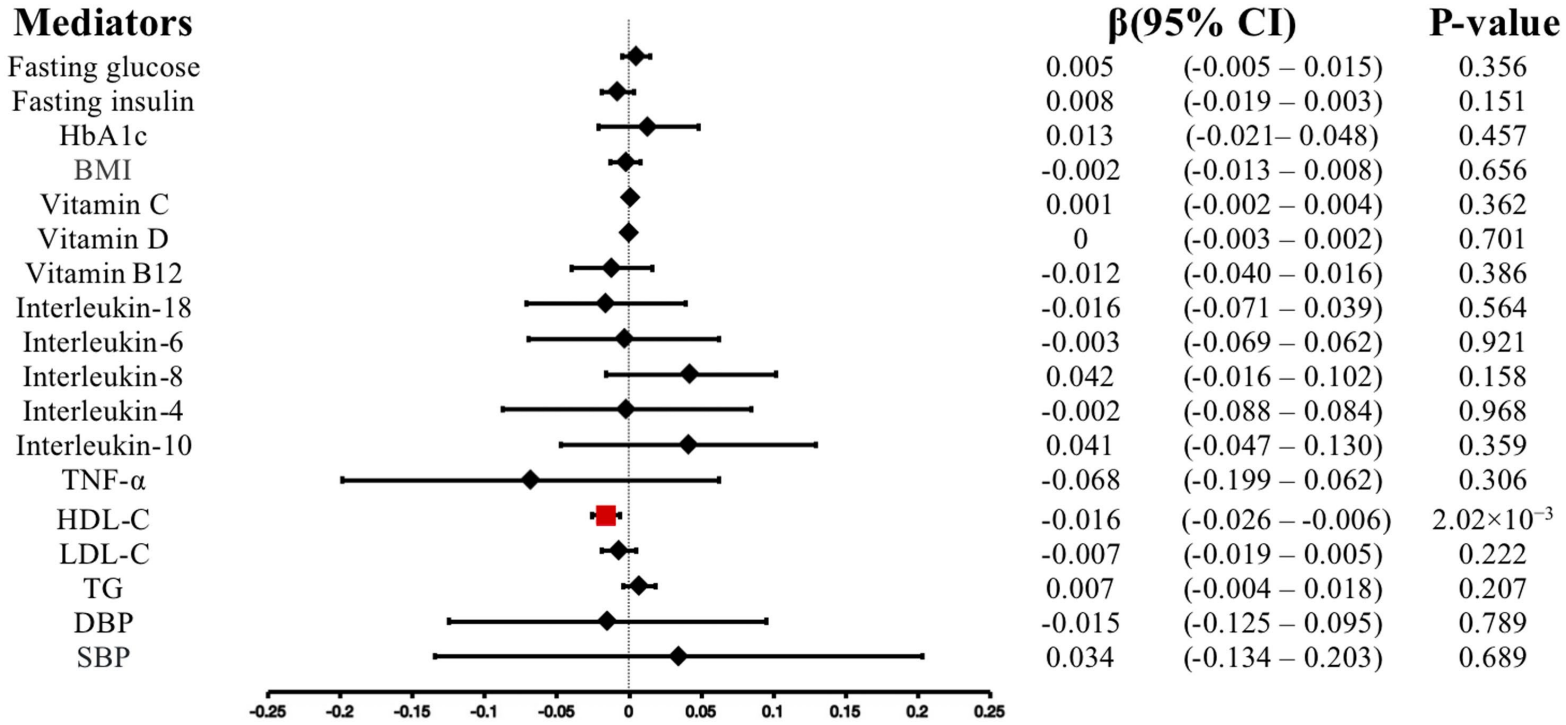
Associations of anti-*H. pylori* IgG levels with mediators. CI, confidence interval; LDL-C, low density lipoprotein cholesterol; HDL-C, high density lipoprotein cholesterol; TG, triglyceride; BMI, body mass index, TNF-α, Tumor necrosis factor alpha; DBP, diastolic blood pressure; SBP, systolic blood pressure.

We conducted MR-PRESSO analysis to detect some outliers with pleiotropy. Then we conduct MR estimates. In this study, we found that the increase of anti-*H. pylori* IgG levels is associated with the decrease of HDL cholesterol levels (*β*, −0.016; 95% CI, −0.026 to −0.006; *p* = 2.02 × 10^−3^). Similar results were obtained using IVW analyses under random effect model (*β*, −0.016; 95% CI, −0.026 to −0.006; *p* = 2.02 × 10^−3^). Using the weighted median method, identical risk estimates were obtained as well (β, −0.018; 95% CI, −0.032 to −0.004; p = 0.011) (Figure 3A). We found no evidence of causal relationship between anti-*H. pylori* IgG levels and LDL cholesterol levels (*β*, −0.007; 95% CI, −0.019 to 0.005; *p* = 0.222), triglyceride (*β*, 0.007; 95% CI, −0.004 to 0.018; *p* = 0.207), fasting glucose (*β*, 0.005; 95% CI, −0.005– 0.015; *p* = 0.356), fasting insulin (*β*, −0.008; 95% CI, −0.019 to 0.003; *p* = 0.151), HbA1c (*β*, 0.013; 95% CI, −0.021– 0.048; *p* = 0.457), BMI (*β*, −0.002; 95% CI, −0.013– 0.008; *p* = 0.656), vitamin C (*β*, 0.001; 95% CI, −0.002 to 0.004; *p* = 0.362), vitamin D (*β*, 0; 95% CI, −0.003 to 0.002; *p* = 0.701), vitamin B12 (*β*, −0.012; 95% CI, −0.040 to 0.016; *p* = 0.386), interleukin-18 (*β*, −0.016; 95% CI, −0.071 to 0.039; *p* = 0.564), interleukin-6 (*β*, −0.003; 95% CI, −0.069 to 0.062; *p* = 0.921), interleukin-8 (*β*, 0.042; 95% CI, −0.016 to 0.102; *p* = 0.158), interleukin-4 (*β*, −0.002; 95% CI, −0.088 to 0.084; *p* = 0.968), interleukin-10 (*β*, 0.041; 95% CI, −0.047 to 0.130; *p* = 0.359), TNF-α (*β*, −0.068; 95% CI, −0.199 to 0.062; *p* = 0.306), diastolic blood pressure (*β*, −0.015; 95% CI, −0.125 to 0.095; *p* = 0.789), systolic blood pressure (*β*, 0.034; 95% CI, −0.134 to 0.203; *p* = 0.689) (Figure 4). To verify the robustness of the causal relationship between anti-*H. pylori* IgG levels and HDL cholesterol levels, we also performed sensitivity analyses. Leave-one-out sensitivity analysis demonstrated that the MR estimations were not driven by a single SNP (Figure 3B). The approach of MR-Egger regression did not reveal any horizontal pleiotropy by the intercept, which indicated that exposure is less likely to affect the outcome through confounders. (intercept = −0.003, SE = 0.005, *p* = 0.336) (Figure 3C). Finally, no heterogeneity was found among studies, as demonstrated by the *p* value of the Cochran Q test (Figure 3D). Details of the MR estimates and sensitivity analyses can be found in the Supplementary material.

## Discussion

In recent years, researchers have focused on the relationship between *H. pylori* infection and MI, but due to residual confounding and reverse causation, these studies have difficulty identifying causality conclusively. MR is founded on the assumption that genetic variations in humans occur at random in the population, are sufficiently independent of confounders, and can be identified as instrumental variables to evaluate the causal relationship between exposure and outcome (Emdin et al., 2017). In this study, we first discovered direct proof showing the causal relationship between anti-*H. pylori* IgG levels and MI using MR analysis. Moreover, we revealed the causality of anti-*H. pylori* IgG levels on HDL cholesterol levels. We concluded that increased anti-*H. pylori* IgG levels are associated with increased risks of MI in the European population, which might be explained by lower HDL cholesterol levels.

A case–control study conducted by Azarkar et al., including 78 individuals with no history of heart disease and 73 MI patients, revealed that a significant difference in *H. pylori* IgG levels was shown between cases and controls (*p* = 0.002) (Azarkar et al., 2011). The study consisting of 100 consecutive patients verified to have suffered acute myocardial infarction, conducted by Kahan et al. (2000), confirmed prior research linking *H. pylori* seropositivity to an increased risk of MI. A meta-analysis conducted by Rahmani et al. (2017) also reported that *H. pylori* infection increased the occurrence of MI. There are two potential assumptions for the significant relationship between *H. pylori* and the risk of MI. First of all, *H. pylori* deoxyribonucleic acid was discovered in the aortic tissue and atherosclerotic plaque of individuals with ischemic heart disease, according to the study by Reszka et al. This finding suggests a direct involvement for bacteria in the pathophysiology of ischemic heart disease and, by extension, MI (Reszka et al., 2008). Secondly, *H. pylori* infection may lower HDL cholesterol levels and raise triglyceride levels. In addition, there might be an elevation in blood levels of coagulation markers and inflammatory factors, including fragments of prothrombin and fibrinogen, tumour necrosis factor, and interleukin 6 and 8. These factors might contribute to the relationship between *H. pylori* and ischemic diseases (Kowalski, 2001).

Notably, our research reveals the causal relationship between anti-*H. pylori* IgG levels and HDL cholesterol, in accordance with a cross-sectional study composed of 961 patients showing that a strong link between *H. pylori* seropositivity and HDL cholesterol (Jia et al., 2009). Hoffmeister et al. (2001) conducted a study that recruited 470 healthy blood donors and 238 patients, observing that HDL cholesterol concentration was substantially reduced in HP-positive (1.36 vs. 1.44 mmol/L, *p* = 0.006) healthy participants compared to negatives. After multivariable adjustment, the effect of *H. pylori* infection on decreased HDL cholesterol (*p* = 0.002) remained notably. A study conducted by Gunji et al. (2008) showed that *H. pylori* seropositivity was significantly relate to lower HDL cholesterol levels. A large cross-sectional study conducted by Kim et al. (2016) showed that *H. pylori* infection was significantly connected with lower HDL-C levels (coefficient = −1.237, *p* < 0.001). Similarly, a prospective, open-label, single-centre study that consisted of 159 patients indicated that patients with *H. pylori* infection had significantly lower levels of HDL cholesterol compared to those without *H. pylori* infection (*p* < 0.05) (Gen et al., 2010). This conclusion is reinforced further by our current analysis.

There is no obvious explanation for how *H. pylori* infection reduces HDL cholesterol levels. Research showed that lipopolysaccharides from *H. pylori* induce the production of inflammatory cytokines in the host, such as tumour necrosis factor-α (TNF-α), interleukin-1, and interleukin-6 (Manolakis et al., 2007). The increase of these inflammatory cytokines may cause *H. pylori* infection to interfere with lipid metabolism (Feingold and Grunfeld, 1992; Georges et al., 2003), that results in atherosclerosis in *H. pylori*-infected individuals (Chen et al., 2016). Lipoprotein lipase activity is actually weakened by TNF-α (Makoveichuk et al., 1862), which causes the transfer of lipids from the tissue to lower levels of HDL cholesterol (Kucukazman et al., 2009). Furthermore, IL-6 and TNF-α disrupt lipid metabolism by boosting the production of liver cholesterol. Future research is required to determine the potential mechanism between *H. pylori* infection and HDL cholesterol.

This study is the first to examine the causality of anti-*H. pylori* IgG levels on MI and potential pathogenesis using MR analyses. Our results are consistent with those from conventional observational research, showing that elevated anti-*H. pylori* IgG levels are significantly related to an increased risk of MI (OR, 1.104; 95% CI, 1.042–1.169; *p* = 7.084 × 10^−4^) and decreases in HDL cholesterol levels (*β*, −0.016; 95% CI, −0.026 to −0.006; *p* = 2.02 × 10^−3^). This study provides a valuable basis for the prevention of CHD patients. There are several advantages of our MR research. To begin, we utilized MR analyses to examine the causality of anti-*H. pylori* IgG levels on MI and HDL cholesterol levels, supplementing the inadequacies of conventional observational studies and providing new evidence for causal relationship between chronic infection and the development of CHD. Second, we used multiple independent SNPs as genetic variants to lessen the influence of linkage disequilibrium on possible relationships. Third, we performed a variety of MR analysis methods and conducted comprehensive sensitivity analyses to verify our findings. Like with other MR investigations, our research also had several limitations that should be taken into account. To begin with, our research was conducted on Europeans, so it’s not known if the results generalize to other populations. Secondly, when we selected IVs, we used a more lenient threshold (*p* < 5 × 10^−6^). Although this may boost statistical power, the more instrumental variables included in the study, the greater the possibility of introducing multi-effect instrumental variables. In order to eliminate horizontal pleiotropy, we conducted sensitivity analyses such as the MR-Egger intercept, MR-PRESSO, and leave-one-out analysis. However, it is very difficult to completely exclude directional pleiotropy because SNPs affect exposure and outcome by independent approach, which has decreased the reliability of the findings. Finally, there is a distinction between *H. pylori* seropositivity and actual persistent infection, since either a false-negative or a false-positive result cannot be ruled out entirely, which may overstate the relationship between the bacteria and the MI.

## Conclusion

At the genetic level, our study provides evidence supporting the causality of anti-*H. pylori* IgG levels on MI and HDL cholesterol levels. Increased anti-*H. pylori* IgG levels are significantly associated with an increased risk of MI and decreases in HDL cholesterol levels. Further clinical research is needed to confirm whether early *H. pylori* eradication can decrease the risk of MI.

## Data availability statement

The original contributions presented in the study are included in the article/Supplementary material, further inquiries can be directed to the corresponding authors.

## Author contributions

QW: Writing – original draft, Conceptualization, Methodology. YL: Writing – review & editing, Visualization. ZX: Writing – review & editing, Data curation. ZW: Writing – review & editing, Data curation. MX: Writing – review & editing. XL: Writing – original draft, Validation. YW: Writing – review & editing, Supervision, Project administration.

## Funding

The author(s) declare that no financial support was received for the research, authorship, and/or publication of this article.

## Conflict of interest

The authors declare that the research was conducted in the absence of any commercial or financial relationships that could be construed as a potential conflict of interest.

## Publisher’s note

All claims expressed in this article are solely those of the authors and do not necessarily represent those of their affiliated organizations, or those of the publisher, the editors and the reviewers. Any product that may be evaluated in this article, or claim that may be made by its manufacturer, is not guaranteed or endorsed by the publisher.
