# Supplementary material for: Causality of anti-Helicobacter pylori IgG levels on myocardial infarction and potential pathogenesis: a Mendelian randomization study
Source: Front Microbiol. 2023 Sep 14;14:1259579. doi: 10.3389/fmicb.2023.1259579 (PMC10538966; doi:10.3389/fmicb.2023.1259579)
Supplement: Supplementary file 1 [file Data_Sheet_1.pdf]

Mendelian randomization estimates between anti-H. pylori IgG titer and Myocardial infarction.

Outcomes	No. of SNPs	Ivw (Random effect)		Ivw (Fixed effect)		Weighted median		Mr-Egger	
		OR (95%CI)	P-value	OR (95%CI)	P-value	OR (95%CI)	P-value	OR(95%CI)	P-value
Myocardial infarction	11	1.104 1.034–1.178	3.116×10^{-3}	1.104 (1.042–1.169)	7.084×10^{-4}	1.178 (1.029–1.214)	8.134×10^{-3}	1.017 (0.851–1.215)	0.857

Mendelian randomization estimates between anti-H. pylori IgG titer and HDL cholesterol.

Outcomes	No. of SNPs	Ivw (Random effect)		Ivw (Fixed effect)		Weighted median		Mr-Egger	
		β(95%CI)	P-value	β(95%CI)	P-value	β(95%CI)	P-value	β(95%CI)	P-value
HDL cholesterol	11	-0.016 (-0.026–-0.006)	2.02×10^{-3}	-0.016 (-0.026–-0.006)	2.02×10^{-3}	-0.018 (-0.032–-0.004)	0.011	-0.001 (-0.031– 0.029)	0.931

Sensitive analyses for the Mendelian randomization analysis between anti-Helicobacter pylori IgG levels and Myocardial infarction

Outcomes	Pleiotropy test(outliers-corrected)	Heterogeneity test(outliers-corrected)			Outliers
	MR-Egger intercept test p value	Cochran's Q	Degrees of Freedom	Cochran's Q p value	
Myocardial infarction	0.358	13.122	10	0.217	rs117912702

Sensitive analyses for the Mendelian randomization analysis between anti-Helicobacter pylori IgG levels and mediators.

Outcomes	Pleiotropy test(outliers-corrected)	Heterogeneity test(outliers-corrected)			Outliers
	MR-Egger intercept test p value	Cochran's Q	Degrees of Freedom	Cochran's Q p value	
Fasting glucose	0.193	10.825	11	0.458	NA
Fasting insulin	0.313	12.178	11	0.350	NA
HbA1c	0.328	6.040	10	0.812	NA
BMI	0.560	12.318	10	0.264	Rs35030589
Vitamin C	0.418	11.490	11	0.403	NA
Vitamin D	0.534	20.609	10	0.024	NA
VitaminB12	0.813	10.009	11	0.529	NA
Interleukin-18	0.120	10.127	11	0.519	NA
Interleukin-6	0.261	15.564	11	0.158	NA
Interleukin-8	0.970	15.219	11	0.173	NA
Interleukin-4	0.543	5.215	9	0.815	NA
Interleukin-10	0.760	6.319	9	0.708	NA
TNF-α	0.332	4.362	9	0.886	NA
HDL	0.336	7.832	10	7.832	Rs 2169557
LDL	0.586	3.780	8	0.876	Rs7502937
					Rs2169557
					Rs35030589
TG	0.179	11.165	10	0.345	Rs35030589
Diastolic blood pressure	0.186	9.960	7	0.191	Rs12591869
					Rs35030589
					Rs55871438
Systolic blood pressure	0.450	15.577	9	0.076	NA

Genome-wide significant SNPs for anti-H. pylori IgG titer

SNP	Chr	Pos	EA/OA	EAF	SNP-Exposure (anti-H. pylori IgG titer)			SNP-Outcome (MI)			F
					Beta	SE	p	Beta	SE	p	
rs12591869	15	96674267	A/C	0.27	-0.13	0.03	1.29E-06	-0.036	0.012	0.003	30.70
rs17502937	13	30440740	T/G	0.02	-0.40	0.08	2.89E-06	-0.038	0.038	0.320	29.11
rs2169557	2	20244954	T/C	0.49	-0.11	0.02	4.55E-06	-0.019	0.011	0.071	26.39
rs35030589	6	32672903	A/G	0.13	-0.18	0.03	3.40E-07	0.008	0.015	0.580	33.16
rs41263973	1	32162810	A/G	0.03	0.32	0.07	2.74E-06	0.056	0.031	0.075	30.45
rs55871438	15	75411120	C/T	0.04	0.30	0.06	4.02E-06	0.002	0.028	0.950	31.95
rs72708546	4	170153219	A/G	0.06	-0.23	0.05	1.90E-06	-0.042	0.023	0.063	27.63
rs73512476	11	86585900	T/G	0.08	0.21	0.04	1.47E-06	-0.011	0.020	0.590	30.17
rs74045808	14	34457396	T/C	0.11	-0.17	0.04	4.49E-06	-0.027	0.018	0.120	27.57
rs77516628	4	62125618	T/A	0.09	0.19	0.04	2.94E-06	0.004	0.019	0.850	26.93
rs78825412	15	83512621	A/C	0.03	0.32	0.07	3.64E-06	0.044	0.031	0.160	29.86

SNP	Chr	Pos	EA/OA	EAF	SNP-Exposure (anti-H. pylori IgG titer)			SNP-Outcome (Fasting glucose)			F
					Beta	SE	p	Beta	SE	p	
rs117912702	6	166062930	A/G	0.02	0.41	0.09	3.02E-06	-0.0082	0.009	0.46	30.22
rs12591869	15	96674267	A/C	0.27	-0.13	0.03	1.29E-06	-0.0012	0.002	0.25	30.70
rs17502937	13	30440740	T/G	0.02	-0.40	0.08	2.89E-06	-0.0021	0.006	0.72	29.11
rs2169557	2	20244954	T/C	0.49	-0.11	0.02	4.55E-06	0.0034	0.002	0.07	26.39
rs35030589	6	32672903	A/G	0.13	-0.18	0.03	3.40E-07	-0.0008	0.003	0.97	33.16
rs41263973	1	32162810	A/G	0.03	0.32	0.07	2.74E-06	0.0014	0.007	0.71	30.45
rs55871438	15	75411120	C/T	0.04	0.30	0.06	4.02E-06	0.0102	0.005	0.04	31.95
rs72708546	4	170153219	A/G	0.06	-0.23	0.05	1.90E-06	-0.0043	0.004	0.54	27.63
rs73512476	11	86585900	T/G	0.08	0.21	0.04	1.47E-06	-0.0002	0.004	0.75	30.17
rs74045808	14	34457396	T/C	0.11	-0.17	0.04	4.49E-06	0.0005	0.003	0.85	27.57
rs77516628	4	62125618	T/A	0.09	0.19	0.04	2.94E-06	0.0045	0.004	0.29	26.93
rs78825412	15	83512621	A/C	0.03	0.32	0.07	3.64E-06	0.0009	0.005	0.88	29.86

SNP	Chr	Pos	EA/OA	EAF	SNP-Exposure (anti-H. pylori IgG titer)			SNP-Outcome (Fasting insulin)			F
					Beta	SE	p	Beta	SE	p	
rs117912702	6	166062930	A/G	0.02	0.41	0.09	3.02E-06	-0.0026	0.011	0.68	30.22
rs12591869	15	96674267	A/C	0.27	-0.13	0.03	1.29E-06	0.0040	0.003	0.21	30.70
rs17502937	13	30440740	T/G	0.02	-0.40	0.08	2.89E-06	-0.0054	0.006	0.38	29.11
rs2169557	2	20244954	T/C	0.49	-0.11	0.02	4.55E-06	-0.0003	0.002	0.68	26.39
rs35030589	6	32672903	A/G	0.13	-0.18	0.03	3.40E-07	0.0086	0.003	0.01	33.16
rs41263973	1	32162810	A/G	0.03	0.32	0.07	2.74E-06	0.0103	0.009	0.08	30.45
rs55871438	15	75411120	C/T	0.04	0.30	0.06	4.02E-06	-0.0032	0.006	0.63	31.95
rs72708546	4	170153219	A/G	0.06	-0.23	0.05	1.90E-06	0.0038	0.004	0.24	27.63

rs73512476	11	86585900	T/G	0.08	0.21	0.04	1.47E-06	0.0023	0.004	0.80	30.17
rs74045808	14	34457396	T/C	0.11	-0.17	0.04	4.49E-06	0.0021	0.004	0.53	27.57
rs77516628	4	62125618	T/A	0.09	0.19	0.04	2.94E-06	-0.0008	0.004	0.74	26.93
rs78825412	15	83512621	A/C	0.03	0.32	0.07	3.64E-06	-0.0038	0.005	0.43	29.86

SNP	Chr	Pos	EA/OA	EAF	SNP-Exposure (anti-H. pylori IgG titer)			SNP-Outcome (HbA1c)			F
					Beta	SE	p	Beta	SE	p	
rs117912702	6	166062930	A/G	0.02	0.41	0.09	3.02E-06	0.021	0.03	0.42	30.22
rs12591869	15	96674267	A/C	0.27	-0.13	0.03	1.29E-06	-0.003	0.01	0.67	30.70
rs17502937	13	30440740	T/G	0.02	-0.40	0.08	2.89E-06	0.009	0.02	0.67	29.11
rs2169557	2	20244954	T/C	0.49	-0.11	0.02	4.55E-06	0.009	0.01	0.16	26.39
rs35030589	6	32672903	A/G	0.13	-0.18	0.03	3.40E-07	-0.005	0.01	0.67	33.16
rs41263973	1	32162810	A/G	0.03	0.32	0.07	2.74E-06	0.011	0.02	0.57	30.45
rs55871438	15	75411120	C/T	0.04	0.30	0.06	4.02E-06	0.019	0.02	0.24	31.95
rs72708546	4	170153219	A/G	0.06	-0.23	0.05	1.90E-06	0.008	0.01	0.57	27.63
rs73512476	11	86585900	T/G	0.08	0.21	0.04	1.47E-06	0.001	0.01	0.92	30.17
rs74045808	14	34457396	T/C	0.11	-0.17	0.04	4.49E-06	-0.013	0.01	0.26	27.57
rs77516628	4	62125618	T/A	0.09	0.19	0.04	2.94E-06	0.014	0.01	0.21	26.93
rs78825412	15	83512621	A/C	0.03	0.32	0.07	3.64E-06	0.005	0.02	0.81	29.86

SNP	Chr	Pos	EA/OA	EAF	SNP-Exposure (anti-H. pylori IgG titer)			SNP-Outcome (BMI)			F
					Beta	SE	p	Beta	SE	p	
rs117912702	6	166062930	A/G	0.02	0.41	0.09	3.02E-06	-0.0097	0.007	0.19	30.22
rs12591869	15	96674267	A/C	0.27	-0.13	0.03	1.29E-06	0.0004	0.002	0.86	30.70
rs17502937	13	30440740	T/G	0.02	-0.40	0.08	2.89E-06	-0.0130	0.007	0.06	29.11
rs2169557	2	20244954	T/C	0.49	-0.11	0.02	4.55E-06	0.0002	0.002	0.93	26.39
rs41263973	1	32162810	A/G	0.03	0.32	0.07	2.74E-06	0.0055	0.006	0.34	30.45
rs55871438	15	75411120	C/T	0.04	0.30	0.06	4.02E-06	0.0022	0.005	0.67	31.95
rs72708546	4	170153219	A/G	0.06	-0.23	0.05	1.90E-06	0.0037	0.004	0.38	27.63
rs73512476	11	86585900	T/G	0.08	0.21	0.04	1.47E-06	0.0033	0.004	0.36	30.17
rs74045808	14	34457396	T/C	0.11	-0.17	0.04	4.49E-06	0.0028	0.003	0.38	27.57
rs77516628	4	62125618	T/A	0.09	0.19	0.04	2.94E-06	-0.0062	0.003	0.07	26.93
rs78825412	15	83512621	A/C	0.03	0.32	0.07	3.64E-06	-0.0041	0.006	0.46	29.86

SNP	Chr	Pos	EA/OA	EAF	SNP-Exposure (anti-H. pylori IgG titer)			SNP-Outcome (Vitamin C)			F
					Beta	SE	p	Beta	SE	p	
rs117912702	6	166062930	A/G	0.02	0.41	0.09	3.02E-06	0.0016	0.002	0.47	30.22
rs12591869	15	96674267	A/C	0.27	-0.13	0.03	1.29E-06	-0.0015	0.001	0.02	30.70
rs17502937	13	30440740	T/G	0.02	-0.40	0.08	2.89E-06	-0.0001	0.002	0.97	29.11
rs2169557	2	20244954	T/C	0.49	-0.11	0.02	4.55E-06	-0.0002	0.001	0.70	26.39
rs35030589	6	32672903	A/G	0.13	-0.18	0.03	3.40E-07	-0.0007	0.001	0.39	33.16

rs41263973	1	32162810	A/G	0.03	0.32	0.07	2.74E-06	-0.0014	0.002	0.40	30.45
rs55871438	15	75411120	C/T	0.04	0.30	0.06	4.02E-06	0.0001	0.002	0.96	31.95
rs72708546	4	170153219	A/G	0.06	-0.23	0.05	1.90E-06	-0.0002	0.001	0.90	27.63
rs73512476	11	86585900	T/G	0.08	0.21	0.04	1.47E-06	0.0013	0.001	0.23	30.17
rs74045808	14	34457396	T/C	0.11	-0.17	0.04	4.49E-06	0.0014	0.001	0.14	27.57
rs77516628	4	62125618	T/A	0.09	0.19	0.04	2.94E-06	-0.0010	0.001	0.32	26.93
rs78825412	15	83512621	A/C	0.03	0.32	0.07	3.64E-06	0.0010	0.002	0.56	29.86

SNP	Chr	Pos	EA/OA	EAF	SNP-Exposure (anti-H. pylori IgG titer)			SNP-Outcome (Vitamin D)			F
					Beta	SE	p	Beta	SE	p	
rs12591869	15	96674267	A/C	0.27	-0.13	0.03	1.29E-06	-0.00089	0.0005	0.05	30.70
rs17502937	13	30440740	T/G	0.02	-0.40	0.08	2.89E-06	0.00128	0.0014	0.36	29.11
rs2169557	2	20244954	T/C	0.49	-0.11	0.02	4.55E-06	0.00004	0.0004	0.91	26.39
rs41263973	1	32162810	A/G	0.03	0.32	0.07	2.74E-06	-0.00117	0.0006	0.04	30.45
rs55871438	15	75411120	C/T	0.04	0.30	0.06	4.02E-06	0.00084	0.0012	0.47	31.95
rs72708546	4	170153219	A/G	0.06	-0.23	0.05	1.90E-06	-0.00133	0.0010	0.21	27.63
rs73512476	11	86585900	T/G	0.08	0.21	0.04	1.47E-06	0.00053	0.0009	0.54	30.17
rs74045808	14	34457396	T/C	0.11	-0.17	0.04	4.49E-06	-0.00025	0.0007	0.73	27.57
rs77516628	4	62125618	T/A	0.09	0.19	0.04	2.94E-06	0.00162	0.0007	0.01	26.93
rs78825412	15	83512621	A/C	0.03	0.32	0.07	3.64E-06	-0.00108	0.0007	0.13	29.86

SNP	Chr	Pos	EA/OA	EAF	SNP-Exposure (anti-H. pylori IgG titer)			SNP-Outcome (Vitamin B12)			F
					Beta	SE	p	Beta	SE	p	
rs117912702	6	166062930	A/G	0.02	0.41	0.09	3.02E-06	-0.0165	0.02	0.41	30.22
rs12591869	15	96674267	A/C	0.27	-0.13	0.03	1.29E-06	0.0038	0.01	0.53	30.70
rs17502937	13	30440740	T/G	0.02	-0.40	0.08	2.89E-06	0.0251	0.02	0.19	29.11
rs2169557	2	20244954	T/C	0.49	-0.11	0.02	4.55E-06	0.0002	0.01	0.97	26.39
rs35030589	6	32672903	A/G	0.13	-0.18	0.03	3.40E-07	0.0036	0.01	0.65	33.16
rs41263973	1	32162810	A/G	0.03	0.32	0.07	2.74E-06	0.0356	0.02	0.02	30.45
rs55871438	15	75411120	C/T	0.04	0.30	0.06	4.02E-06	-0.0165	0.01	0.25	31.95
rs72708546	4	170153219	A/G	0.06	-0.23	0.05	1.90E-06	-0.0043	0.01	0.71	27.63
rs73512476	11	86585900	T/G	0.08	0.21	0.04	1.47E-06	-0.0045	0.01	0.66	30.17
rs74045808	14	34457396	T/C	0.11	-0.17	0.04	4.49E-06	-0.0047	0.01	0.60	27.57
rs77516628	4	62125618	T/A	0.09	0.19	0.04	2.94E-06	-0.0048	0.01	0.61	26.93
rs78825412	15	83512621	A/C	0.03	0.32	0.07	3.64E-06	-0.0099	0.02	0.52	29.86

SNP	Chr	Pos	EA/OA	EAF	SNP-Exposure (anti-H. pylori IgG titer)			SNP-Outcome (Interleukin-18)			F
					Beta	SE	p	Beta	SE	p	
rs117912702	6	166062930	A/G	0.02	0.41	0.09	3.02E-06	0.043	0.04	0.31	30.22
rs12591869	15	96674267	A/C	0.27	-0.13	0.03	1.29E-06	0.017	0.01	0.19	30.70
rs17502937	13	30440740	T/G	0.02	-0.40	0.08	2.89E-06	0.033	0.03	0.33	29.11

rs2169557	2	20244954	T/C	0.49	-0.11	0.02	4.55E-06	0.017	0.01	0.10	26.39
rs41263973	1	32162810	A/G	0.03	0.32	0.07	2.74E-06	0.002	0.02	0.88	30.45
rs55871438	15	75411120	C/T	0.04	0.30	0.06	4.02E-06	0.029	0.04	0.41	31.95
rs72708546	4	170153219	A/G	0.06	-0.23	0.05	1.90E-06	-0.001	0.03	0.97	27.63
rs73512476	11	86585900	T/G	0.08	0.21	0.04	1.47E-06	0.028	0.02	0.22	30.17
rs74045808	14	34457396	T/C	0.11	-0.17	0.04	4.49E-06	0.004	0.02	0.84	27.57
rs77516628	4	62125618	T/A	0.09	0.19	0.04	2.94E-06	0.003	0.02	0.89	26.93
rs78825412	15	83512621	A/C	0.03	0.32	0.07	3.64E-06	0.023	0.02	0.21	29.86

SNP	Chr	Pos	EA/OA	EAF	SNP-Exposure (anti-H. pylori IgG titer)			SNP-Outcome (Interleukin-6)			F
					Beta	SE	p	Beta	SE	p	
rs117912702	6	166062930	A/G	0.02	0.41	0.09	3.02E-06	-0.01	0.05	0.92	30.22
rs12591869	15	96674267	A/C	0.27	-0.13	0.03	1.29E-06	-0.01	0.02	0.40	30.70
rs17502937	13	30440740	T/G	0.02	-0.40	0.08	2.89E-06	-0.03	0.04	0.42	29.11
rs2169557	2	20244954	T/C	0.49	-0.11	0.02	4.55E-06	0.01	0.01	0.29	26.39
rs41263973	1	32162810	A/G	0.03	0.32	0.07	2.74E-06	0.04	0.02	0.02	30.45
rs55871438	15	75411120	C/T	0.04	0.30	0.06	4.02E-06	0.07	0.04	0.11	31.95
rs72708546	4	170153219	A/G	0.06	-0.23	0.05	1.90E-06	0.02	0.03	0.63	27.63
rs73512476	11	86585900	T/G	0.08	0.21	0.04	1.47E-06	0.05	0.03	0.04	30.17
rs74045808	14	34457396	T/C	0.11	-0.17	0.04	4.49E-06	0.01	0.03	0.63	27.57
rs77516628	4	62125618	T/A	0.09	0.19	0.04	2.94E-06	-0.01	0.03	0.84	26.93
rs78825412	15	83512621	A/C	0.03	0.32	0.07	3.64E-06	0.01	0.02	0.73	29.86

SNP	Chr	Pos	EA/OA	EAF	SNP-Exposure (anti-H. pylori IgG titer)			SNP-Outcome (Interleukin-8)			F
					Beta	SE	p	Beta	SE	p	
rs117912702	6	166062930	A/G	0.02	0.41	0.09	3.02E-06	0.001	0.04	0.98	30.22
rs12591869	15	96674267	A/C	0.27	-0.13	0.03	1.29E-06	-0.033	0.01	0.02	30.70
rs17502937	13	30440740	T/G	0.02	-0.40	0.08	2.89E-06	-0.017	0.04	0.63	29.11
rs2169557	2	20244954	T/C	0.49	-0.11	0.02	4.55E-06	0.005	0.01	0.64	26.39
rs41263973	1	32162810	A/G	0.03	0.32	0.07	2.74E-06	0.018	0.02	0.29	30.45
rs55871438	15	75411120	C/T	0.04	0.30	0.06	4.02E-06	0.100	0.04	0.01	31.95
rs72708546	4	170153219	A/G	0.06	-0.23	0.05	1.90E-06	0.016	0.03	0.60	27.63
rs73512476	11	86585900	T/G	0.08	0.21	0.04	1.47E-06	0.022	0.02	0.36	30.17
rs74045808	14	34457396	T/C	0.11	-0.17	0.04	4.49E-06	0.012	0.02	0.59	27.57
rs77516628	4	62125618	T/A	0.09	0.19	0.04	2.94E-06	-0.016	0.02	0.46	26.93
rs78825412	15	83512621	A/C	0.03	0.32	0.07	3.64E-06	0.017	0.02	0.41	29.86

SNP	Chr	Pos	EA/OA	EAF	SNP-Exposure (anti-H. pylori IgG titer)			SNP-Outcome (Interleukin-4)			F
					Beta	SE	p	Beta	SE	p	
rs12591869	15	96674267	A/C	0.27	-0.13	0.03	1.29E-06	-0.009	0.02	0.65	30.70
rs17502937	13	30440740	T/G	0.02	-0.40	0.08	2.89E-06	-0.013	0.04	0.78	29.11

rs2169557	2	20244954	T/C	0.49	-0.11	0.02	4.55E-06	0.014	0.02	0.38	26.39
rs41263973	1	32162810	A/G	0.03	0.32	0.07	2.74E-06	0.002	0.02	0.90	30.45
rs55871438	15	75411120	C/T	0.04	0.30	0.06	4.02E-06	0.076	0.06	0.20	31.95
rs72708546	4	170153219	A/G	0.06	-0.23	0.05	1.90E-06	-0.017	0.04	0.65	27.63
rs73512476	11	86585900	T/G	0.08	0.21	0.04	1.47E-06	-0.035	0.03	0.27	30.17
rs74045808	14	34457396	T/C	0.11	-0.17	0.04	4.49E-06	-0.029	0.04	0.44	27.57
rs77516628	4	62125618	T/A	0.09	0.19	0.04	2.94E-06	0.012	0.04	0.89	26.93
rs78825412	15	83512621	A/C	0.03	0.32	0.07	3.64E-06	-0.004	0.03	0.91	29.86

SNP	Chr	Pos	EA/OA	EAF	SNP-Exposure (anti-H. pylori IgG titer)			SNP-Outcome (Interleukin-10)			F
					Beta	SE	p	Beta	SE	p	
rs12591869	15	96674267	A/C	0.27	-0.13	0.03	1.29E-06	-0.015	0.02	0.47	30.70
rs17502937	13	30440740	T/G	0.02	-0.40	0.08	2.89E-06	-0.029	0.04	0.50	29.11
rs2169557	2	20244954	T/C	0.49	-0.11	0.02	4.55E-06	-0.018	0.02	0.27	26.39
rs41263973	1	32162810	A/G	0.03	0.32	0.07	2.74E-06	0.011	0.02	0.55	30.45
rs55871438	15	75411120	C/T	0.04	0.30	0.06	4.02E-06	0.062	0.06	0.31	31.95
rs72708546	4	170153219	A/G	0.06	-0.23	0.05	1.90E-06	-0.041	0.04	0.32	27.63
rs73512476	11	86585900	T/G	0.08	0.21	0.04	1.47E-06	-0.028	0.03	0.39	30.17
rs74045808	14	34457396	T/C	0.11	-0.17	0.04	4.49E-06	-0.022	0.04	0.57	27.57
rs77516628	4	62125618	T/A	0.09	0.19	0.04	2.94E-06	-0.001	0.04	0.95	26.93
rs78825412	15	83512621	A/C	0.03	0.32	0.07	3.64E-06	-0.006	0.03	0.85	29.86

SNP	Chr	Pos	EA/OA	EAF	SNP-Exposure (anti-H. pylori IgG titer)			SNP-Outcome (TNF-α)			F
					Beta	SE	p	Beta	SE	p	
rs12591869	15	96674267	A/C	0.27	-0.13	0.03	1.29E-06	-0.012	0.03	0.71	30.70
rs17502937	13	30440740	T/G	0.02	-0.40	0.08	2.89E-06	-0.027	0.06	0.67	29.11
rs2169557	2	20244954	T/C	0.49	-0.11	0.02	4.55E-06	0.025	0.02	0.31	26.39
rs35030589	6	32672903	A/G	0.13	-0.18	0.03	3.40E-07	0.035	0.03	0.19	33.16
rs41263973	1	32162810	A/G	0.03	0.32	0.07	2.74E-06	0.047	0.09	0.61	30.45
rs55871438	15	75411120	C/T	0.04	0.30	0.06	4.02E-06	0.012	0.06	0.87	31.95
rs72708546	4	170153219	A/G	0.06	-0.23	0.05	1.90E-06	0.031	0.05	0.52	27.63
rs73512476	11	86585900	T/G	0.08	0.21	0.04	1.47E-06	-0.069	0.06	0.23	30.17
rs74045808	14	34457396	T/C	0.11	-0.17	0.04	4.49E-06	0.002	0.06	0.91	27.57
rs77516628	4	62125618	T/A	0.09	0.19	0.04	2.94E-06	-0.004	0.05	0.93	26.93
rs78825412	15	83512621	A/C	0.03	0.32	0.07	3.64E-06	-0.018	0.07	0.76	29.86

SNP	Chr	Pos	EA/OA	EAF	SNP-Exposure (anti-H. pylori IgG titer)			SNP-Outcome (HDL)			F
					Beta	SE	p	Beta	SE	p	
rs117912702	6	166062930	A/G	0.02	0.41	0.09	3.02E-06	0.0002	0.007	0.98	30.22
rs12591869	15	96674267	A/C	0.27	-0.13	0.03	1.29E-06	0.0013	0.002	0.54	30.70
rs17502937	13	30440740	T/G	0.02	-0.40	0.08	2.89E-06	-0.0050	0.007	0.45	29.11

rs35030589	6	32672903	A/G	0.13	-0.18	0.03	3.40E-07	0.0072	0.003	0.01	33.16
rs41263973	1	32162810	A/G	0.03	0.32	0.07	2.74E-06	-0.0060	0.005	0.28	30.45
rs55871438	15	75411120	C/T	0.04	0.30	0.06	4.02E-06	-0.0086	0.005	0.08	31.95
rs72708546	4	170153219	A/G	0.06	-0.23	0.05	1.90E-06	0.0013	0.004	0.75	27.63
rs73512476	11	86585900	T/G	0.08	0.21	0.04	1.47E-06	-0.0045	0.004	0.21	30.17
rs74045808	14	34457396	T/C	0.11	-0.17	0.04	4.49E-06	0.0042	0.003	0.17	27.57
rs77516628	4	62125618	T/A	0.09	0.19	0.04	2.94E-06	-0.0023	0.003	0.50	26.93
rs78825412	15	83512621	A/C	0.03	0.32	0.07	3.64E-06	-0.0063	0.005	0.24	29.86

SNP	Chr	Pos	EA/OA	EAF	SNP-Exposure (anti-H. pylori IgG titer)			SNP-Outcome (LDL)			F
					Beta	SE	p	Beta	SE	p	
rs117912702	6	166062930	A/G	0.02	0.41	0.09	3.02E-06	-0.003	0.008	0.71	30.22
rs12591869	15	96674267	A/C	0.27	-0.13	0.03	1.29E-06	0.001	0.002	0.55	30.70
rs41263973	1	32162810	A/G	0.03	0.32	0.07	2.74E-06	-0.008	0.006	0.20	30.45
rs55871438	15	75411120	C/T	0.04	0.30	0.06	4.02E-06	-0.008	0.005	0.12	31.95
rs72708546	4	170153219	A/G	0.06	-0.23	0.05	1.90E-06	-0.002	0.004	0.68	27.63
rs73512476	11	86585900	T/G	0.08	0.21	0.04	1.47E-06	-0.001	0.004	0.80	30.17
rs74045808	14	34457396	T/C	0.11	-0.17	0.04	4.49E-06	0.001	0.003	0.84	27.57
rs77516628	4	62125618	T/A	0.09	0.19	0.04	2.94E-06	0.002	0.004	0.54	26.93
rs78825412	15	83512621	A/C	0.03	0.32	0.07	3.64E-06	-0.002	0.006	0.73	29.86

SNP	Chr	Pos	EA/OA	EAF	SNP-Exposure (anti-H. pylori IgG titer)			SNP-Outcome (TG)			F
					Beta	SE	p	Beta	SE	p	
rs117912702	6	166062930	A/G	0.02	0.41	0.09	3.02E-06	0.012	0.007	0.11	30.22
rs12591869	15	96674267	A/C	0.27	-0.13	0.03	1.29E-06	-0.002	0.002	0.45	30.70
rs17502937	13	30440740	T/G	0.02	-0.40	0.08	2.89E-06	-0.004	0.007	0.54	29.11
rs2169557	2	20244954	T/C	0.49	-0.11	0.02	4.55E-06	-0.004	0.002	0.06	26.39
rs41263973	1	32162810	A/G	0.03	0.32	0.07	2.74E-06	-0.001	0.006	0.80	30.45
rs55871438	15	75411120	C/T	0.04	0.30	0.06	4.02E-06	0.001	0.005	0.78	31.95
rs72708546	4	170153219	A/G	0.06	-0.23	0.05	1.90E-06	0.004	0.004	0.29	27.63
rs73512476	11	86585900	T/G	0.08	0.21	0.04	1.47E-06	0.002	0.004	0.54	30.17
rs74045808	14	34457396	T/C	0.11	-0.17	0.04	4.49E-06	-0.005	0.003	0.13	27.57
rs77516628	4	62125618	T/A	0.09	0.19	0.04	2.94E-06	0.000	0.003	0.96	26.93
rs78825412	15	83512621	A/C	0.03	0.32	0.07	3.64E-06	-0.008	0.006	0.16	29.86

SNP	Chr	Pos	EA/OA	EAF	SNP-Exposure (anti-H. pylori IgG titer)			SNP-Outcome (diastolic pressure)			F
					Beta	SE	p	Beta	SE	p	
rs17502937	13	30440740	T/G	0.02	-0.40	0.08	2.89E-06	-0.066	0.06	0.26	29.11
rs2169557	2	20244954	T/C	0.49	-0.11	0.02	4.55E-06	0.020	0.02	0.25	26.39
rs41263973	1	32162810	A/G	0.03	0.32	0.07	2.74E-06	0.094	0.05	0.08	30.45
rs72708546	4	170153219	A/G	0.06	-0.23	0.05	1.90E-06	-0.004	0.04	0.92	27.63

rs73512476	11	86585900	T/G	0.08	0.21	0.04	1.47E-06	-0.064	0.03	0.05	30.17
rs74045808	14	34457396	T/C	0.11	-0.17	0.04	4.49E-06	0.004	0.03	0.89	27.57
rs77516628	4	62125618	T/A	0.09	0.19	0.04	2.94E-06	0.007	0.03	0.81	26.93
rs78825412	15	83512621	A/C	0.03	0.32	0.07	3.64E-06	-0.032	0.05	0.51	29.86

SNP	Chr	Pos	EA/OA	EAF	SNP-Exposure (anti-H. pylori IgG titer)			SNP-Outcome (systolic pressure)			F
					Beta	SE	p	Beta	SE	p	
rs12591869	15	96674267	A/C	0.27	-0.13	0.03	1.29E-06	-0.058	0.03	0.10	30.70
rs17502937	13	30440740	T/G	0.02	-0.40	0.08	2.89E-06	0.046	0.10	0.64	29.11
rs2169557	2	20244954	T/C	0.49	-0.11	0.02	4.55E-06	0.075	0.03	0.01	26.39
rs35030589	6	32672903	A/G	0.13	-0.18	0.03	3.40E-07	-0.011	0.05	0.80	33.16
rs55871438	15	75411120	C/T	0.04	0.30	0.06	4.02E-06	0.160	0.08	0.06	31.95
rs72708546	4	170153219	A/G	0.06	-0.23	0.05	1.90E-06	-0.015	0.06	0.82	27.63
rs73512476	11	86585900	T/G	0.08	0.21	0.04	1.47E-06	-0.069	0.06	0.23	30.17
rs74045808	14	34457396	T/C	0.11	-0.17	0.04	4.49E-06	-0.057	0.05	0.27	27.57
rs77516628	4	62125618	T/A	0.09	0.19	0.04	2.94E-06	0.017	0.05	0.75	26.93
rs78825412	15	83512621	A/C	0.03	0.32	0.07	3.64E-06	0.002	0.08	0.98	29.86

SNP	Chr	Pos	EA/OA	EAF	SNP-Exposure (anti-H. pylori IgG titer)			SNP-Outcome (systolic pressure)			F
					Beta	SE	p	Beta	SE	p	
rs12591869	15	96674267	A/C	0.27	-0.13	0.03	1.29E-06	-0.058	0.03	0.10	30.70
rs17502937	13	30440740	T/G	0.02	-0.40	0.08	2.89E-06	0.046	0.10	0.64	29.11
rs2169557	2	20244954	T/C	0.49	-0.11	0.02	4.55E-06	0.075	0.03	0.01	26.39
rs35030589	6	32672903	A/G	0.13	-0.18	0.03	3.40E-07	-0.011	0.05	0.80	33.16
rs55871438	15	75411120	C/T	0.04	0.30	0.06	4.02E-06	0.160	0.08	0.06	31.95
rs72708546	4	170153219	A/G	0.06	-0.23	0.05	1.90E-06	-0.015	0.06	0.82	27.63
rs73512476	11	86585900	T/G	0.08	0.21	0.04	1.47E-06	-0.069	0.06	0.23	30.17
rs74045808	14	34457396	T/C	0.11	-0.17	0.04	4.49E-06	-0.057	0.05	0.27	27.57
rs77516628	4	62125618	T/A	0.09	0.19	0.04	2.94E-06	0.017	0.05	0.75	26.93
rs78825412	15	83512621	A/C	0.03	0.32	0.07	3.64E-06	0.002	0.08	0.98	29.86
